# Systematic investigation of the emerging pathogen of *Tsukamurella* species in a Chinese tertiary teaching hospital

**DOI:** 10.1128/spectrum.01644-23

**Published:** 2023-10-24

**Authors:** Shuying Yu, Xiaoqi Ding, Kexin Hua, Huiqing Zhu, Qingshui Zhang, Xinuo Song, Xiuli Xie, Rong Huang, Yingchun Xu, Li Zhang, Qiaolian Yi, Ying Zhao

**Affiliations:** 1 Department of Laboratory Medicine, State Key Laboratory of Complex, Severe, and Rare Diseases, Peking Union Medical College Hospital, Chinese Academy of Medical Sciences, Beijing, China; 2 Beijing Key Laboratory for Mechanisms Research and Precision Diagnosis of Invasive Fungal Diseases, Beijing, China; 3 Department of Clinical Laboratory, Zhangjiajie People’s Hospital, Zhangjiajie, China; 4 Department of Respiratory, Peking Union Medical College Hospital, Chinese Academy of Medical Sciences and Peking Union Medical College, Beijing, China; University of Mississippi Medical Center, Jackson, Mississippi, USA

**Keywords:** antimicrobial susceptibility, identification, *Mycobacterium tuberculosis*, quinolone resistance mechanism, *Tsukamurella*

## Abstract

*Tsukamurella* species have been clinically regarded as rare but emerging opportunistic pathogens causing various infections in humans. *Tsukamurella* pneumonia has often been misdiagnosed as pulmonary tuberculosis due to its clinical presentation resembling tuberculosis-like syndromes. *Tsukamurella* species have also been confused in the laboratory with other phylogenetic bacteria, such as *Gordonia*. This study aimed to investigate the clinical, microbiological, and molecular characteristics; species distribution; and antimicrobial susceptibility of *Tsukamurella* species. Immunodeficiency and chronic pulmonary disease appeared to be risk factors for *Tsukamurella* pneumonia, and the presence of bronchiectasis and pulmonary nodules on imaging was highly correlated with this infection. The study confirmed that *groEL* (heat shock protein 60) and *secA* (the secretion ATPase) genes are reliable for identifying *Tsukamurella* species. Additionally, the *ssrA* (stable small RNA) gene showed promise as a tool for discriminating between different *Tsukamurella* species with the shortest sequence length. In terms of antimicrobial susceptibility, quinolones, trimethoprim/sulfamethoxazole, amikacin, minocycline, linezolid, and tigecycline demonstrated potent *in vitro* activity against *Tsukamurella* isolates in our study. The study also proposed a resistance mechanism involving a substitution (S91R) within the quinolone-resistance-determining region of the *gyrA* gene, which confers resistance to levofloxacin and ciprofloxacin. Furthermore, we found that disk diffusion testing is not suitable for testing the susceptibilities of *Tsukamurella* isolates to ciprofloxacin, imipenem, and minocycline. In conclusion, our systematic investigation may contribute to a better understanding of this rare pathogen.

*Tsukamurella* species are rare but emerging human pathogens that share remarkable similarities with other mycolic acid–containing genera of the order Actinomycetales, especially *Mycobacterium tuberculosis*. Consequently, misdiagnosis and therapeutic failures can occur in clinical settings. Despite the significance of accurate identification, antimicrobial susceptibility, and understanding the resistance mechanism of this important genus, our knowledge in these areas remains fragmentary and incomplete. In this study, we aimed to address these gaps by investigating promising identification methods, the antimicrobial susceptibility patterns, and a novel quinolone resistance mechanism in *Tsukamurella* species, utilizing a collection of clinical isolates. The findings of our study will contribute to improve diagnosis and successful management of infections caused by *Tsukamurella* species, as well as establishing well-defined performance and interpretive criteria for antimicrobial susceptibility testing.

## INTRODUCTION

*Tsukamurella* species are obligate aerobic, Gram-positive, weakly or variably acid-fast, rod-shaped bacillus from the order Actinomycetales (1). These bacteria are environmental saprophytes found in various environments, including soil, water, sludge, and wastewater from petroleum reservoirs (1). Currently, there have been 17 named species within the *Tsukamurella* genus (2, 3). Among these species, 12 species of *Tsukamurella* have been reported to cause a range of human infections, as most reported cases were associated with bacteremia and catheter-related bloodstream infections (CR-BSI) (4 – 12). *Tsukamurella* species have long been attributed to pulmonary infection in immunodeficient patients (3, 13 – 17). *Tsukamurella* pneumonia has been reported to present with symptoms resembling tuberculosis (18), leading to misdiagnosis of pulmonary tuberculosis in some clinical events (13, 16, 19). Additionally, *Tsukamurella* species can cause opportunistic infections such as peritonitis (20, 21), keratitis (22 – 26), conjunctivitis (2, 23, 27, 28), ocular infection (24, 29, 30), meningitis (31), brain abscess (32), knee prosthesis (33), and cutaneous infection (34).

Accurate and rapid identification of the pathogen is crucial for diagnosing and managing infectious diseases. *Tsukamurella* species share many common features with other phylogenetic bacterial groups comprising *Nocardia*, *Gordonia*, *Rhodococcus*, *Corynebacterium*, and *Mycobacterium* (5, 18). *Gordonia*, a genus that bears phenotypical similarity to *Tsukamurella*, has been increasingly isolated in our laboratory and often misidentified as *Tsukamurella* species by conventional phenotypic identification method or matrix-assisted laser desorption ionization–time-of-flight mass spectrometry (MALDI-TOF MS) system. Traditionally, accurately identifying *Tsukamurella* species using conventional phenotypic or biochemical methods in most clinical microbiology laboratories is challenging, leading to the underestimated incidence of *Tsukamurella* infection (35, 36). Several molecular methods have been demonstrated to be comparatively rapid and reliable tools for identifying *Tsukamurella* species, including sequencing of 16S ribosomal RNA (rRNA), the essential secretory protein-encoding gene *secA* (the secretion ATPase), *rpoB* (beta-subunit of DNA-dependent RNA polymerase), *groEL* (60 kDa chaperonin and heat shock protein), and transfer messenger RNA coding gene *ssrA* (stable small RNA) (37 – 39). Among these five gene targets mentioned, the 16S rRNA gene has been considered impractical for distinguishing species within the *Tsukamurella* genus owing to its highly conserved sequence (35). However, it has been found to be sufficiently discriminative in identifying *Tsukamurella* species from phylogenetically related groups (5, 35, 40). Regarding the other four housekeeping genes, Teng et al. recommended that sequencing the *groEL* gene displayed good performance in identifying *Tsukamurella* species at the species level (37). Furthermore, Pérez et al. (41) provided evidence of the *secA1* gene being a useful target for differentiating *Tsukamurella pulmonis* from other clinically significant *Tsukamurella* species. Nevertheless, the discriminatory capacity of these genes warrants further evaluation using a broader range of clinical isolates, encompassing species other than *T. pulmonis* and *Tsukamurella tyrosinosolvens* (37, 41). However, identification by sequencing is still beyond the reach of many routine clinical microbiology laboratories. MALDI-TOF MS has been proven as a potential routine identification tool for *Tsukamurella* species (29). Teng et al. found that the MALDI-TOF MS showed good performance for genus-level identification of 60 *Tsukamurella* isolates using a commercial database but insufficient performance for species-level identification. However, 98.3% of *Tsukamurella* isolates were correctly identified to species level with score ≥2.0 using the expanded in-house database (29). In light of varying antimicrobial susceptibilities among different *Tsukamurella* spp., the continuous expansion of MALDI-TOF MS databases with available spectral data for each *Tsukamurella* species, as well as other phylogenetically related bacteria, would be highly beneficial in enabling accurate, rapid, and cost-effective identification of *Tsukamurella* species in routine clinical laboratories.

To date, the knowledge of well-defined susceptibility or resistance patterns of *Tsukamurella* species is fragmentary and primarily derived from sporadic reports. The Clinical and Laboratory Standards Institute (CLSI) guidelines have recommended the broth microdilution method for antimicrobial susceptibility testing (AST) of aerobic actinomycetes (42). In addition, CLSI M62 has proposed tentative breakpoints for aerobic actinomycetes, which depend mainly upon organism population distributions, clinical data, breakpoints used for other organisms, and experience of experts in the field (43). CLSI has also emphasized the necessity to gather more data for the optimal management of infections caused by aerobic actinomycetes including *Tsukamurella* species (43). The disk diffusion method is easy and convenient for AST of *Nocardia* species and the other aerobic actinomycetes in many clinical laboratories (18, 44). However, well-established interpretive criteria for testing *Tsukamurella* species or other aerobic actinomycetes using the disk diffusion method are lacking. The breakpoints for *Staphylococcus* spp. or Gram-positive bacteria are usually used to perform agar disk assays with Actinomycetales (32, 44, 45). *Tsukamurella* isolates are generally susceptible to amikacin, ciprofloxacin, imipenem, doxycycline, vancomycin, linezolid, and sulfamethoxazole but resistant to penicillin, oxacillin, piperacillin/tazobactam, and cephalosporin (1, 38). Nevertheless, the susceptibility patterns may differ among various *Tsukamurella* spp. (46). Good clinical outcomes and favorable prognoses are highly dependent on accurate identification and appropriate antibiotic therapy with source control (1). Accordingly, it is necessary to clarify the clinical and microbiology features, adequate diagnostic methods, and antimicrobial susceptibility to effectively treat *Tsukamurella* infections.

In the present study, we retrospectively collected clinical isolates that were initially identified as *Tsukamurella* species along with relevant clinical information in the Clinical Microbiology Laboratory of Peking Union Medical College Hospital, Beijing, China, from 2018 to 2022. We aimed to investigate the clinical, microbiological, and molecular characteristics; species distribution; and antimicrobial susceptibility of *Tsukamurella* species.

## MATERIALS AND METHODS

### Patients and bacterial isolates

We analyzed 22 clinical isolates (from 17 patients) of *Tsukamurella* and *Gordonia* species, which were initially recognized as *Tsukamurella* species by the Clinical Microbiology Laboratory of Peking Union Medical College Hospital, Beijing, China, between January 2018 and October 2022. A total of 22 preserved isolates from 17 patients were initially identified as *Tsukamurella* spp. using a MALDI-TOF MS system (Autof MS1000; Autobio Labtec Instruments Co, Ltd., Zhengzhou, China). The samples were prepared as previously described (47), and the identification of isolates was performed using Autof Acquirer V2.0.18 (Autof MS1000, Autobio) following the manufacturer’s criteria for species and genus identification (47). The Autof MS1000 database contained 4 species of *Tsukamurella* and 12 species of *Gordonia*, encompassing *Tsukamurella inchonensis*, *Tsukamurella paurometabola*, *T. pulmonis*, *T. tyrosinosolvens, Gordonia aichiensis*, *Gordonia alkanivorans*, *Gordonia amicalis*, *Gordonia australis*, *Gordonia bronchialis*, *Gordonia humi*, *Gordonia kroppenstedtii*, *Gordonia otitidis*, *Gordonia rubripertincta*, *Gordonia sihwensis*, *Gordonia sputi*, and *Gordonia terrae*. Misidentification referred to discrepant identification results using the MALDI-TOF MS system compared with molecular methods. Chart review focused on 11 patients who were ultimately confirmed to have *Tsukamurella* infection, including 10 cases of probable *Tsukamurella* pneumonia and 1 case of CR-BSI. The patient records were evaluated to determine patient demographics, clinical characteristics of infection (including underlying diseases and pulmonary manifestations), laboratory test results, prior/empirical antibiotic use, therapy, and outcomes. Furthermore, all isolates were thoroughly phenotypically characterized, including observations on colony appearance and cell morphology using Gram stain, Ziehl–Neelsen stain, and modified Ziehl–Neelsen stain.

### Sequence-based identification

For all the isolates, DNA extraction and amplification were performed as previously described (48). The polymerase chain reaction products were sequenced in both directions using the DNA analyzer ABI 3730XL system (Applied Biosystems, CA, USA). Species identification was performed by querying 16S rRNA sequences against those in the GenBank database, with the nucleotide Basic Local Alignment Search Tool (BLASTn, http://blast.ncbi.nlm.nih.gov). Regarding the isolates assigned as *Tsukamurella* species by 16S rRNA gene sequencing, DNA amplification and sequencing of *secA*, *rpoB*, *groEL*, and *ssrA* genes were performed with primer pairs LPW24724/LPW24726, LPW24130/LPW24131, LPW34162/LPW33894, and LPW24733/LPW24730, respectively, as previously described (2, 37). Moreover, the *gyrA* gene from highly quinolone-resistant *Tsukamurella* isolate (22TM00764) and the other quinolone-susceptible *Tsukamurella* isolates were also sequenced using primer pairs GYRA-F (5′-AACTCGAAGGACTTCCATGAC-3′) and GYRA-R (5′- TTCGTGCTCCGCGGCGGCTTA-3′).

### Phylogenetic analysis

The nucleotide sequences of 16S rRNA, *secA*, *rpoB*, and *groEL* for type strains of 16 *Tsukamurella* species and *ssrA* gene for 11 *Tsukamurella* species available in GenBank were included in this study as described by Teng et al. (2, 37). The phylogenetic analysis was performed with Molecular Evolutionary Genetic Analysis software (version 11.0; https://megasoftware.net/) using the neighbor-joining (NJ) method, with all positions containing gaps and missing data eliminated from the data set. The significance of the cluster nodes was determined by bootstrapping with 1,000 randomizations, and the NJ tree was, therefore, generated. The phylogenetic trees were manipulated and adjusted using the online tool The Interactive Tree of Life (https://itol.embl.de) (49). The 16S rRNA, *secA*, *rpoB*, and *groEL* sequences of *Mycobacterium tuberculosis* (GenBank accession no. NR116692; CP074075: 3619823–3620337, CP074075  : 760955–761238, and CP074075: 529466–530142, respectively) and *ssrA* sequences of *Tsukamurella soli* (GenBank accession no. KX932003) were used as outgroups.

### Antimicrobial susceptibility testing

*In vitro* susceptibility was determined using the broth microdilution methodology according to the CLSI M24-3E protocol (42). The minimum inhibitory concentrations (MICs) of 34 tested antibiotics against the 22 isolates were determined and interpreted after 72-h incubation at 37°C (Tables 3 and 4) (43).

Ceftriaxone, amoxicillin/clavulanic acid (2:1), tobramycin, amikacin, minocycline, doxycycline, clarithromycin, vancomycin, imipenem, moxifloxacin, ciprofloxacin, trimethoprim/sulfamethoxazole, linezolid, and rifampin were interpreted with reference to the breakpoints for testing *Nocardia* spp. and other aerobic actinomycetes (43). Besides, the interpretation of cefoxitin and meropenem referred to the breakpoints for rapidly growing mycobacteria, whereas the interpretation of ethambutol and isoniazid referred to the *M. tuberculosis* complex (43). No breakpoints or interpretive criteria were available for some of the antimicrobial agents tested; therefore, only an MIC was reported without interpretation.

We routinely used the disk diffusion method to perform AST on 15 antimicrobial disks (Table 5) for aerobic actinomycetes on Mueller–Hinton agar with 5% sheep blood. The inocula were prepared according to the CLSI M24-3E protocol (42). The results of the inhibition zone diameter were recorded after 72-h incubation at 37°C and interpreted based on the thresholds adapted from the guidelines of the Antibiogram Committee of the French Society of Microbiology devoted to Gram-positive bacteria (50) and CLSI guidelines devoted to *Staphylococcus aureus* (51) (Table S1). *S. aureus* (ATCC 29213) and *Escherichia coli* (ATCC 35218) were used for quality control.

### Nucleotide sequence accession numbers

The 16S rRNA, *secA*, *rpoB*, *groEL*, and *ssrA* gene sequences of 15 isolates assigned as *Tsukamurella* spp. were deposited in GenBank with accession numbers as shown in Fig. 2. Meanwhile, the 16S rRNA gene sequences of eight isolates assigned as *Gordonia* spp. were also deposited in GenBank (Table S2).

## RESULTS

### Clinical characteristics

A total of 15 isolates identified as *Tsukamurella* spp. were recognized as pathogens causing probable pulmonary infection in 10 patients (12 isolates) and CR-BSI in 1 patient (three isolates). The clinical characteristics of probable pulmonary infection and CR-BSI caused by *Tsukamurella* spp. in 11 patients are summarized in Tables 1 and 2, respectively. Of 10 patients with probable *Tsukamurella* pulmonary infection, 7 were aged ≥60 years, and 1 was a teenager. Out of 10 patients, 7 were female. Six patients received steroid administration, yet five patients had an immune disease and the other four patients had chronic lung disease. All the patients with probable *Tsukamurella* pulmonary infection presented with associated airway constitutional symptoms; pulmonary nodules on imaging seemed to suggest this kind of infection. In addition, 4 out of 10 patients had bronchiectasis, whereas other pathogens were isolated in 6 patients, including *Pseudomonas aeruginosa*, *S. aureus*, *Enterobacter cloacae*, and *Pneumocystis jirovecii*, at the same time. Tuberculosis was clinically suspected in five patients. Unfortunately, three patients had been on anti-tuberculosis therapy for years as an empirical diagnosis of negative tuberculosis. In particular, patient 10 was diagnosed with tuberculosis in a local hospital on the basis of a sputum smear showing acid-fast bacilli. While the misdiagnosis was discovered by sputum culture, it was positive for *T. pulmonis* in 2020 and for *T. inchonensis* in 2018 and 2019 but negative for mycobacterium tuberculosis culture. Five patients showed good improvement after treatment with levofloxacin, sulfamethoxazole, or trimethoprim. Furthermore, a 51-year-old woman presented to the general surgical department complaining of intermittent abdominal distension with vomiting for 3 months (Table 2). She underwent surgery for a mesentery tumor on 29 December 2021. Bacteremia caused by *T. tyrosinosolvens* was diagnosed in this patient with CR-BSI in the presence of a peripherally inserted central catheter (PICC) on 12 January 2022. The patient had a good clinical outcome after ciprofloxacin and amikacin therapy (Table 2).

**TABLE 1:** Clinical characteristics of 10 patients with probable pulmonary infection caused by *Tsukamurella* species^*a*^

Patients	1	2	3	4	5	6	7	8	9	10
Demographics (sex/age/)	87/M	56/F	64/F	63/F	73/F	80/F	63/M	63/F	12/M	53/F
Inpatient/outpatient	Outpatient	Outpatient	Outpatient	Inpatient	Outpatient	Inpatient	Outpatient	Outpatient	Inpatient	Outpatient
Reasons for seeking medical support	Pulmonary nodules	Cough for 4 months	Cough, expectoration, hemoptysis, and shortness of breath	Cough and expectoration for 50 years, aggravated with wheezing and decreased activity tolerance	Bronchiectasis	Severe pneumonia	Pulmonary nodules	Cough for 5 months	Chest pain and hemoptysis for 1 month	Pulmonary nodules and hydropericardium
Date of admission	6 September 2022	13 October 2022	15 June 2022	9 March 2022	14 November 2021	9 March 2021	1 April 2021	5 June 2020	9 January 2019	12 January 2018
Department of admission	Respiratory medicine	Respiratory medicine	Respiratorymedicine	Cardiology	Respiratory medicine	Emergency	Respiratory medicine	Respiratory medicine	Immunology	Respiratory medicine
Strain no.	22TM02858	22NC06989	22TM01897	22TM00764	21TM04007	21TM01062	21TM00974	20TM00845	19TP0049	18TM0123/19TM0-2205/20TM02422
Initial identification by MALDI-TOF MS^*a*^	*T. paurometabola*	*T. pulmonis*	*T. paurometa-bola*	*T. paurometa-bola*	*T. paurometabola*	*T. paurometabola*	*T. paurometabola*	*T. paurometabola*	*T. paurometabola*	*T. inchonensis/T. inchonensis/T. paurometabola*
Identification by gene sequencing	*T. tyrosinosolvens*	*T. sputi*	*T. pulmonis*	*T. tyrosinosolvens*	*T. tyrosinosolvens*	*T. pulmonis*	*T. tyrosinosolvens*	*T. tyrosinosolvens*	*T. tyrosinosolvens*	*T. inchonensis/T. inchonensis/T. pulmonis*
Site of isolation	Sputum	Sputum	Sputum	Sputum	Sputum	Sputum	Sputum	Sputum	Sputum	Sputum
Date of isolation	10 October 2022	22 October 2022	8 August 2022	22 March 2022	15 December 2021	24 May 2021	8 May 2021	23 June 2020	15 March 2019	9 March 2018/23 July 2019/16 December 2020
Underlying disease	Hypertension, COPD, DM, and M-proteinemia	GERD, anaphylactic rhinitis	Hydronephrosis, bronchiectasis, and senile vaginitis	Pulmonary hypertension, respiratory failure, hypertension, and hyperlipidemia	RA, SS, lung adenocarcinoma after surgery	ITP, DM, hyperlipemia, hypertension,	No	SLE	SLE	IgG4-RD and hypertension
Bronchiectasis	No	No	Yes	Yes	Yes	No	Yes	No	No	No
Pulmonary manifestations	Cough and sputum, asthma, and pulmonary nodules	Cough and sputum, hemoptysis, and nodules in the pulmonary cavity	Cough, expectoration, hemoptysis, shortness of breath, and multiple small and faint nodules in the pulmonary cavity	Cough and sputum, respiratory failure, and respiratory acidosis	Cough and purulent phlegm with blood	Chest tightness, dyspnea, decrease in activity tolerance, and pulmonary nodules	Cough, hemoptysis, and pulmonary nodules	Cough, purulent phlegm with blood, and pulmonary nodules	Chest pain, cough, and hemoptysis	Cough and pulmonary nodules
Clinical suspicion of tuberculosis	No	Yes	No	Yes	No	No	Yes	Yes	No	Yes
Isolation of other pathogens	No	No	*Pseudomonas aeruginosa*(sputum)	*P. aeruginosa* (sputum)	*Staphylococcus aureus* (sputum)	*Pneumocystis jirovecii* (sputum)	No	*P. aeruginosa* (sputum)	*P. aeruginosa*, *Enterobacter cloacae* (sputum)	No
Immune disease	No	No	No	No	Yes	Yes	No	Yes	Yes	Yes
Use of steroids	No	No	Yes	No	Yes	Yes	No	Yes	Yes	Yes
Surgery	No	No	No	No	Yes	No	No	No	No	No
Prior/empiricalantibiotic usage	No	Isoniazid, ethambutol, and pyrazinamide	Acetylcysteine	Moxifloxacin and levofloxacin	Rifapentine, isoniazid, and ethambutol	Trimethoprim and moxifloxacin	Moxifloxacin	Moxifloxacin	Ertapenem and sulfamethoxazole	Rifapentine and isoniazid
Therapy after culture	Acetylcysteine	Unknown	Unknown	NA (discharged)	Levofloxacin	Sulfamethoxazole	Unknown	Trimethoprim	NA (discharged)	Levofloxacin and sulfamethoxazole
Outcome	No improvement	Loss to follow-up	Loss to follow-up	Recover	Recover	Recover	Loss to follow-up	Recover	Recover	Recover

^*a*^ COPD, chronic obstructive pulmonary disease; DM, diabetes mellitus; GERD, gastroesophageal reflux disease; IgG4-RD, IgG4-related diseases; ITP, immune thrombocytopenia; MALDI-TOF MS, matrix-assisted laser desorption ionization–time-of-flight mass spectrometry; NA, not available; RA, rheumatoid arthritis; SLE, systemic lupus erythematosus; SS, Sjögren’s syndrome.

**TABLE 2:** Clinical characteristics of one patient with CR-BSI caused by *T. tyrosinosolvens^a^*

Patients	11
Demographics (sex/age)	51/F
Inpatient/outpatient	Inpatient
Reasons for seeking medical support	Intermittent bloating with vomiting for 3 months
Date of admission	27 December 2021
Department of admission	General surgery
Date of discharge	17 January 2022
Strain no.	22B00890/22B00957/22W00319
Initial identification by MALDI-TOF MS^*a*^	*T. paurometabola*
Identification by gene sequencing	*T. tyrosinosolvens*
Site of isolation	Blood, PICC catheter blood, and PICC catheter
Date of isolation	12 January 2022/14 January 2022
Underlying disease	Cancer
Clinical status at the time of positive culture	
Immunosuppressive state	Yes
Surgery within 30 days	Yes, partial resection of small intestine
Fever	Yes
CRP	Elevated
Pulmonary comorbidities	Pulmonary infection
Presence of PICC/drainage tube	Yes
Parenteral nutrition	Yes
Prior/empirical antibiotic use	Moxifloxacin
Concomitant infection	No
Therapy after culture	Ciprofloxacin and amikacin
Outcomes	Recovery

^*a*^ CRP, C-reactive protein; MALDI-TOF MS, matrix-assisted laser desorption ionization–time-of-flight mass spectrometry; PICC, peripherally inserted central catheter.

### Species identification

Of 22 clinical isolates initially identified as *Tsukamurella* spp. by the MALDI-TOF MS system, 15 isolates from 11 patients were confirmed as *Tsukamurella* spp. by 16S rRNA, *secA*, *rpoB*, *groEL*, and *ssrA* gene sequencing. *T. tyrosinosolvens* (*n* = 9) was the most prevalent pathogen, followed by *T. pulmonis* (*n* = 3), *T. inchonensis* (*n* = 2), and *T. sputi* (*n* = 1) (Tables 1 and 2). *T. tyrosinosolvens* and *T. pulmonis* were misidentified as *T. paurometabola* and *T. sputi* as *T. pulmonis* under the MALDI-TOF MS platform, whereas *T. inchonensis* was correctly identified. Besides, seven other isolates obtained from six patients were identified as *Gordonia* spp. by 16S rRNA gene sequencing, including *G. sputi* (*n* = 4), *G. aichiensis* (*n* = 2), and *G. bronchialis* (*n* = 1) (Table S2). These seven *Gordonia* isolates were all misidentified as *T. pulmonis* (five isolates), *T. inchonensis* (one isolates), or *Tsukamurella* spp. (one isolate, Table S2) by MALDI-TOF MS. Moreover, 16S rRNA gene sequencing showed sufficient discriminatory power for intergeneric identification of the genera *Tsukamurella* and *Gordonia* but not for intraspecies identification of the genus *Tsukamurella* along.

### Phenotypic characterization

**Fig 1:**
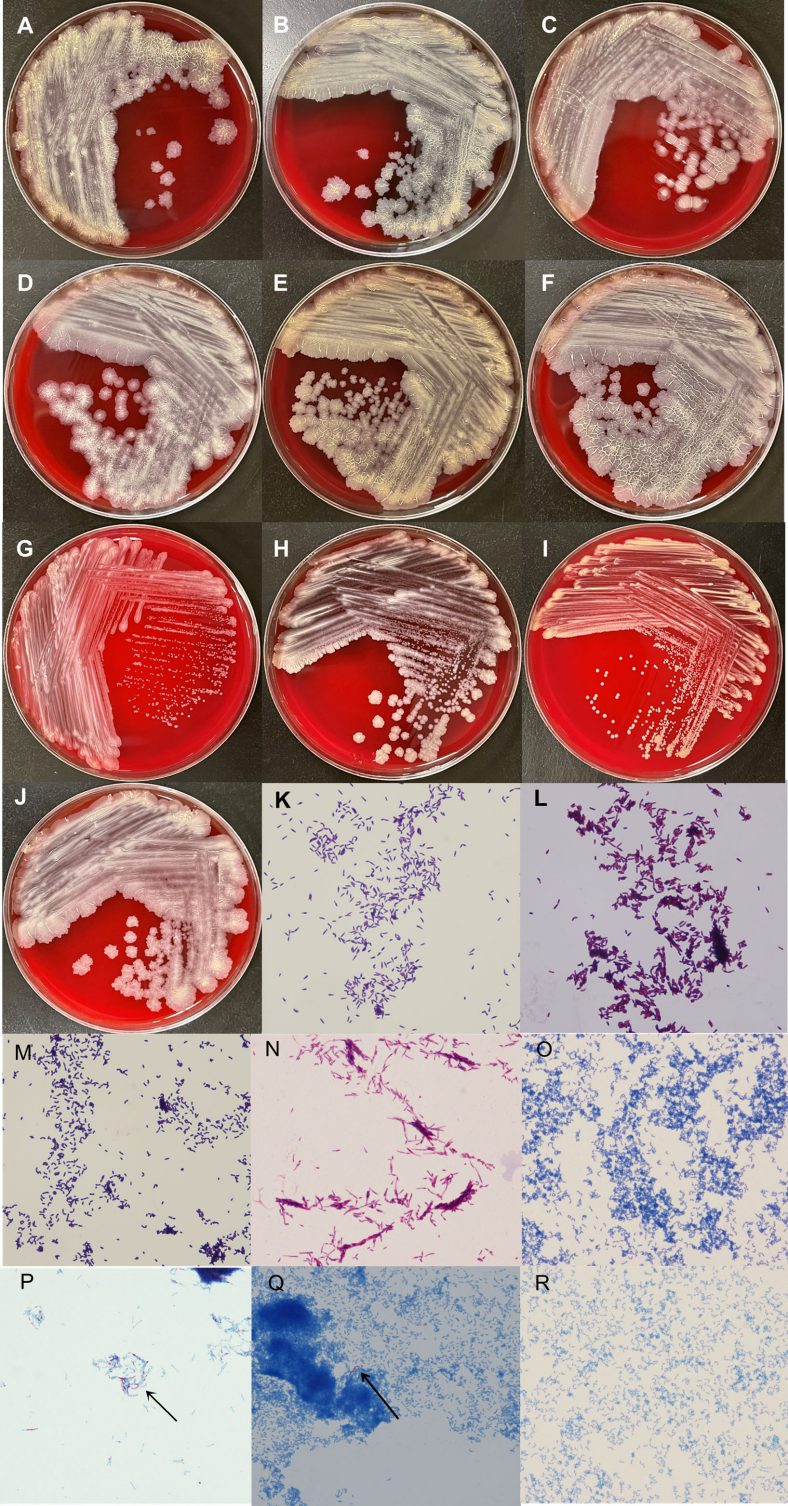
Colony appearance and cell morphology results of *Tsukamurella and Gordonia* isolates. Note: the isolates were grown on Columbia blood agar and incubated at 37°C for 48 h (A–J). Rod-shaped organisms shown by Gram staining (**K–M**), modified Ziehl–Neelsen staining (**N–O**), and Ziehl–Neelsen staining (**P–R**). (**A and B**) *T. tyrosinosolvens* (22B00957 and 19TP0049), (**C and D**) *T. pulmonis* (22TM01897 and 20TM02422), (**E**) *T. inchonensis* (18TM0123), (**F**) *T. sputi* (22NC06989), (**G**) *G. sputi* (21NC9120), (**H**) *G. sputi* (21NC03851), (**I**) *G. aichiensis* (21NC05575), and (**J**) *G. bronchialis* (19W04084). (**K**) *T. pulmonis* (20TM02422), (**L**) *T. tyrosinosolvens* (22B00890), (**M**) *G. bronchialis* (19W04084), (**N**) *T. tyrosinosolvens* (22B00957), (**O**) *G. aichiensis* (21NC05575), (**P**) *T. inchonensis* (18TM0123), (**Q**) *T. sputi* (22NC06989), and (**R**) *G. aichiensis* (21NC05575).

The colonies of *Tsukamurella* isolates were white-grayish to yellow colored, dry, wrinkled, and resembled a rough membrane with irregular spreading fringes on Columbia blood agar plates after 48-h incubation at 37°C under aerobic conditions (Fig. 1A through F). *G. bronchialis* displayed white-grayish creamy colored, dry, and membrane-like colonies with irregular spreading fringes, similar to *Tsukamurella* isolates (Fig. 1J). Besides, the other *Gordonia* isolates exhibited white-grayish to yellow colored but smooth, butyrous, and circumscribed small colonies under the same condition (Fig. 1G through I). The colony appearance of the same *Tsukamurella* or *Gordonia* species may show different characteristics, yet different *Tsukamurella* and *Gordonia* species have similarities (Fig. 1A through J). Gram staining showed Gram-positive, slender, rod-shaped bacilli for *Tsukamurella* isolates or slightly short and thick cocci–rod bacilli for *Gordonia* isolates (Fig. 1K through M). The results of modified Ziehl–Neelsen staining (Fig. 1N) and Ziehl–Neelsen staining (Fig. 1P and Q) were positive and partially positive for *Tsukamurella* isolates, respectively. Ziehl–Neelsen staining and modified Ziehl–Neelsen staining results were negative for the *Gordonia* isolates in this study (Fig. 1O and R).

### Phylogenetic analysis

**Fig 2:**
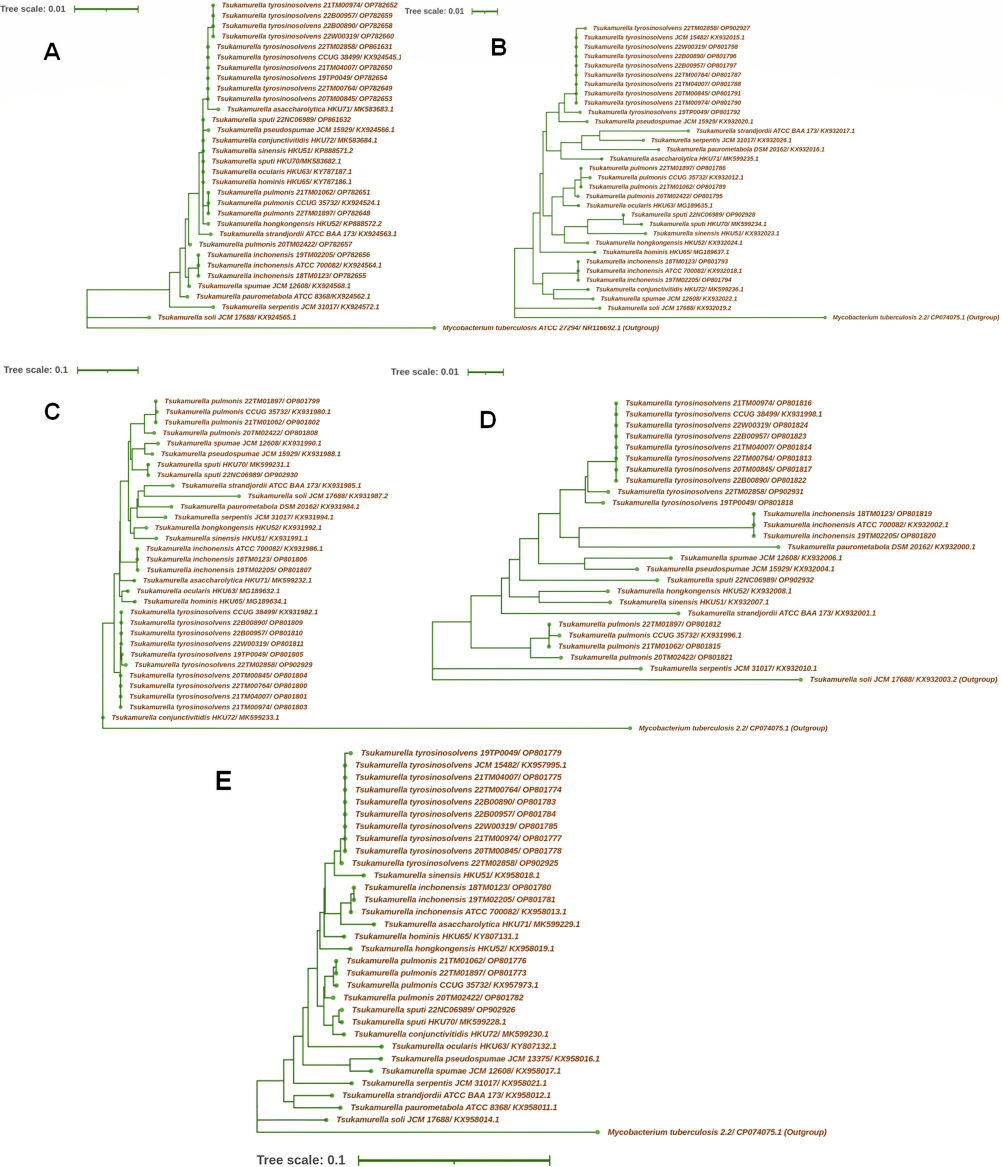
Phylogenetic trees generated using the NJ method based on the nucleotide sequences of (**A**) 16S rRNA, (**B**) *rpoB*, (**C**) *secA*, (**D**) *ssrA*, and (**E**) *groEL* from this study and nucleotide sequences of 16S rRNA, *secA*, *rpoB*, and *groEL* for type strains of 16 *Tsukamurella* species, while *ssrA* gene for type strains of 11 *Tsukamurella* species were available in GenBank. The 16S rRNA, *secA*, *rpoB*, and *groEL* sequences of *M. tuberculosis* (GenBank accession no. NR116692; CP074075: 13619823–3620337, CP074075: 1760955–761238, and CP074075: 1529466–530142, respectively) and *ssrA* sequence of *Tsukamurella soli* (GenBank accession no. KX932003) were used as outgroups. Names, strain numbers, and accession numbers are given as cited in the GenBank database. The scale bar indicates the estimated number of substitutions per base.

A total of 16 *Tsukamurella* species (16S rRNA, *secA*, *rpoB*, and *groEL*) or 12 *Tsukamurella* species (*ssrA* gene) were employed to construct phylogenetic trees (Fig. 2). Phylogenetic trees were inferred from (A) partial 16S rRNA (1,221 nucleotide positions), (B) partial *rpoB* (284 nucleotide positions), (C) partial *secA* (607 nucleotide positions), (D) partial *ssrA* (366 nucleotide positions), and (E) partial *groEL* (677 nucleotide positions) gene sequence data using the NJ method (Fig. 2). Comparative 16S rRNA gene sequence analysis revealed a low sequence variability among species of the genus *Tsukamurella* (Fig. 2A). Some recently recognized novel species in the genus *Tsukamurella*, including *T. sputi*, *T. conjunctivitidis*, *T. sinensis*, *T. ocularis*, and *T. hominis*, could not be distinguished by 16S rRNA gene. The other four housekeeping genes showed higher variation than the 16S rRNA region. Each individual housekeeping gene assembled in the 15 *Tsukamurella* isolates (four species) in our study formed monophyletic clusters with the corresponding *Tsukamurella* type strain but exhibited intraspecies polymorphisms. Isolate 20TM02422 was the most isolated compared with other isolates in the *T. pulmonis* species in the above five loci of the phylogenetic tree, especially for the 16S rRNA gene. The *T. tyrosinosolvens* isolates 19TP0049 and 22TM02858 showed a more distant relationship with the other *T. tyrosinosolvens* isolates in four loci of the phylogenetic tree (except for 16S rRNA gene, Fig. 2). No reference *ssrA* sequence of *T. sputi* is available in the National Center for Biotechnology Information (NCBI) database. The *ssrA* sequence of *T. sputi* isolate 22NC06989 in this study was submitted with the accession number OP902932. Compared with the phylogenetic tree constructed from all five loci, the *ssrA* gene had the highest intraspecies discrimination with the shortest sequence length in the 12 closely related species of the genus *Tsukamurella*. Unfortunately, no *ssrA* sequences were available in the NCBI database for investigating the regional divergence of the four *Tsukamurella* species, including *T. asaccharolytica*, *T. hominis*, *T. conjunctivitidis*, and *T. ocularis*.

### Antimicrobial susceptibilities

We observed differentiate antimicrobial susceptibilities between *Tsukamurella* and *Gordonia* to ceftazidime, cefoxitin, and amoxicillin/clavulanic acid (2:1) in this study. The MIC value of all the *Tsukamurella* isolates to ceftazidime was >32 µg/mL, whereas the MIC range of *Gordonia* isolates was 4–8 µg/mL. All the *Tsukamurella* isolates showed high MICs to cefoxitin and amoxicillin clavulanic acid (2:1), which were >128 and >64/32 µg/mL, respectively. However, all the *Gordonia* isolates showed higher susceptibility to cefoxitin and amoxicillin/clavulanic acid (2:1) with the MIC of 16 and ≤2/1 µg/mL, respectively. All the *Tsukamurella* and *Gordonia* isolates were susceptible to amikacin and linezolid with MIC values of ≤1–2 µg/mL. Except for one *T. tyrosinosolvens* isolate (21TM00974), which was intermediate to minocycline with an MIC of 2 µg/mL, the other *Tsukamurella* and all *Gordonia* isolates were susceptible to minocycline with an MIC of ≤1 µg/mL. The MIC values of all the *Tsukamurella* and *Gordonia* isolates against daptomycin were more than 2 µg/mL, while the MIC range against tigecycline was 0.12–0.5 µg/mL. Regarding obligate anti-tuberculosis agents, including isoniazid, ethionamide, and ethambutol, all the *Tsukamurella* and *Gordonia* isolates showed high resistance with MIC values of more than 8 µg/mL, more than 20 µg/mL, and 16 µg/mL, respectively. Overall, the *Gordonia* isolates showed higher susceptibilities than the *Tsukamurella* isolates to penicillin, chloramphenicol, aminoglycosides, tetracyclines, and macrolides (Tables 3 and 4). A clear differentiation between *Tsukamurella* and *Gordonia* spp. was also observed for antimicrobial susceptibilities to vancomycin (Tables 3 and 4). For carbapenems, only two *T. pulmonis* isolates (22TM01897 and 21TM01062) were not susceptible to imipenem and meropenem in this study (Table 3). One *T. tyrosinosolvens* isolate (22TM00764) demonstrated extremely high resistance to levofloxacin and ciprofloxacin but intermediate to moxifloxacin (Table 3). The other isolates in this study were susceptible to quinolones. Trimethoprim/sulfamethoxazole appeared to be a potent antimicrobial agent with strong *in vitro* antimicrobial activity (Tables 3 and 4). Moreover, rifampin was not a good choice against infection caused by *Tsukamurella* and *Gordonia* because of high non-susceptibility (Tables 3 and 4).

**TABLE 3:** Results of antimicrobial susceptibility testing using broth microdilution methodology among *Tsukamurella* isolates according to species^*a*^

Antimicrobial agents	*Tsukamurella* spp. (*n* = 15)		*T. tyrosinosolvens* (*n* = 9)		*T. pulmonis* (*n* = 3)		*T. inchonensis* (*n* = 2)		*T. sputi* (*n* = 1)	
	MIC range	*N* (%) of NS	MIC range	*N* (%) of NS	MIC range	*N* (%) of NS	MIC range	*N* (%) of NS	MIC range	***N*** (%) of NS
Penicillin	≥4	NA	>4	NA	>4	NA	4	NA	>4	NA
Cefuroxime	2 to >4	NA	2 to >4	NA	>4	NA	2 to 2	NA	>4	NA
Ceftriaxone	0.5 to 4	0	1 to 2	0	1	0	0.5 to 1	0	4	0
Cefotaxime	0.5 to 4	NA	0.5 to 2	NA	0.5 to 1	NA	1 to 2	NA	4	NA
Cefepime	≤1 to 8	NA	≤1 to 4	NA	4	NA	≤1 to 2	NA	8	NA
Streptomycin	8 to >64	NA	32 to 64	NA	8 to 32	NA	>64	NA	16	NA
Tobramycin	2 to 16	10 (66.67)	4 to 16	8 (88.89)	2	0	4 to 8	1 (50)	8	1 (100)
Doxycycline	0.5 to 8	13 (86.67)	0.5 to 4	8 (88.89)	1 to 4	2 (66.67)	8	2 (100)	4	1 (100)
Tetracycline	4 to >8	NA	4 to >8	NA	8 to >8	NA	>8	NA	>8	NA
Chloramphenicol	16 to >32	NA	32	NA	16 to >32	NA	>32	NA	32	NA
Erythromycin	>2	NA	>2	NA	>2	NA	>2	NA	>2	NA
Azithromycin	2 to >2	NA	>2	NA	>2	NA	>2	NA	2	NA
Clarithromycin	0.12 to 4	6 (40)	2–4	4 (44.44)	1 to 2	0	4	2 (100)	0.12	0
Clindamycin	>1	NA	>1	NA	>1	NA	>1	NA	>1	NA
Vancomycin	4 to 16	15 (100)	8 to 16	9 (100)	4	3 (100)	16	2 (100)	16	1 (100)
Ertapenem	2 to >4	NA	≥4	NA	≥4	NA	4	NA	2	NA
Imipenem	0.5 to 16	2 (13.33)	1 to 2	0	1 to 16	2 (66.67)	0.5	0	0.5	0
Meropenem	2 to 16	2 (13.33)	2 to 4	0	2 to 16	2 (66.67)	2	0	2	0
Levofloxacin	≤0.5 to >4	NA	≤0.5 to >4	NA	≤0.5	NA	≤0.5	NA	≤0.5	NA
Moxifloxacin	≤0.12 to 2	1 (6.67)	≤0.12 to 2	1 (11.11)	≤0.12	0	0.25	0	≤0.12	0
Ciprofloxacin	≤0.12 to >8	1 (6.67)	0.5 to >8	1 (11.11)	0.25	0	0.5	0	≤0.12	0
Trimethoprim/sulfamethoxazole	≤0.12/2.38 to 0.25/4.75	0	≤0.12/2.38 to 0.25/4.75	0	≤0.12/2.38 to 0.25/4.75	0	0.25/4.75	0	0.25/4.75	0
Rifampin	1 to 4	12 (80)	1 to 4	8 (88.89)	1 to 2	1 (33.33)	4	2 (100)	2	1 (100)
Rifabutin	1 to 8	NA	4 to 8	NA	1 to 2	NA	8	NA	4	NA

^*a*^ MIC, Minimum inhibitory concentration; NA, not available; NS, non-susceptible.

**TABLE 4:** Results of antimicrobial susceptibility testing using broth microdilution methodology among *Gordonia* isolates according to species^*a*^

Antimicrobial agents	*Gordonia* spp. ( = 7)		*G. sputi* (*n* = 4)		*G. aichiensis* (*n* = 2)		*G. Bronchialis* (*n* = 1)	
	MIC range	*N* (%) of NS	MIC range	*N* (%) of NS	MIC range	*N* (%) of NS	MIC range	*N* (%) of NS
Penicillin	0.25 to 2	NA	0.5 to 1	NA	0.25 to 1	NA	2	NA
Cefuroxime	2 to 4	NA	2 to 4	NA	4	NA	2	NA
Ceftriaxone	1 to 2	0	1 to 2	0	1	0	1	0
Cefotaxime	0.5 to 1	NA	0.5 to 1	NA	0.5	NA	0.5	NA
Cefepime	≤1	NA	≤1	NA	≤1	NA	≤1	NA
Streptomycin	≤0.5 to 16	NA	1 to 16	NA	≤0.5 to 16	NA	16	NA
Tobramycin	≤1	0	≤1	0	≤1	0	≤1	0
Doxycycline	≤0.12 to 0.5	0	0.25 to 0.5	0	≤0.12 to 0.5	0	0.5	0
Tetracycline	4 to 8	NA	4 to 8	NA	4 to 8	NA	8	NA
Chloramphenicol	8 to 16	NA	8 to 16	NA	8 to 16	NA	16	NA
Erythromycin	≤0.25 to >2	NA	≤0.25 to >2	NA	0.5 to 1	NA	2	NA
Azithromycin	≤0.25 to >2	NA	≤0.25 to >2	NA	1	NA	>2	NA
Clarithromycin	≤0.06 to 2	0	≤0.06 to 2	0	0.12 to 0.25	0	1	0
Clindamycin	0.5 to 1	NA	0.5 to 1	NA	1	NA	1	NA
Vancomycin	0.5 to 1	0	1	0	0.5	0	1	0
Ertapenem	1 to 4	NA	one to 2	NA	1 to 4	NA	2	NA
Imipenem	≤0.25	0	≤0.25	0	≤0.25	0	≤0.25	0
Meropenem	0.5 to 1	0	0.5 to 1	0	0.5	0	0.5	0
Levofloxacin	≤0.5	NA	≤0.5	NA	≤0.5	NA	≤0.5	NA
Moxifloxacin	≤0.12 to 0.25	0	≤0.12 to 0.25	0	≤0.12	0	≤0.12	0
Ciprofloxacin	≤0.12	0	≤0.12	0	≤0.12	0	≤0.12	0
Trimethoprim/sulfamethoxazole	≤0.12/2.38 to 0.25/4.75	0	≤0.12/2.38 to 0.25/4.75	0	≤0.12/2.38 to 0.25/4.75	0	0.25/4.75	0
Rifampin	≤0.12 to >8	4 (57.14)	≤0.12 to >8	3 (75)	≤0.12	0	4	1 (100)
Rifabutin	≤0.25 to 8	NA	≤0.25 to 8	NA	≤0.25	NA	4	NA

^*a*^ MIC, Minimum inhibitory concentration; NA, not available; NS, non-susceptible.

Based on the results of AST by disk diffusion method, the interpretation categories of all *Tsukamurella* and *Gordonia* isolates to amoxicillin/clavulanic acid, linezolid, and trimethoprim/sulfamethoxazole demonstrated 100% agreement with the results of the broth microdilution method in this study, with complete resistance to amoxicillin/clavulanic acid in *Tsukamurella* isolates, complete susceptibility in *Gordonia* isolates, and all susceptibility to linezolid and trimethoprim/sulfamethoxazole irrespective of *Tsukamurella* and *Gordonia* isolates (Tables 3 to 5). Conversely, the disk diffusion method for ceftriaxone susceptibility showed a 60% of susceptibility against *Tsukamurella* isolates compared with 100% susceptibility by the broth microdilution method (Tables 3 and 5). In addition, completely opposite interpretation categories to cefoxitin for *Gordonia* isolates and vancomycin for *Tsukamurella* isolates were found between these two methods (Tables 3 to 5). The disk diffusion method failed to detect the ciprofloxacin resistant isolate 22TM00764 with an inhibitory zone diameter of 38 mm. Although the inhibitory zone diameter of isolates 22TM00764 to moxifloxacin was 24 mm, it was still interpreted as susceptible based on the breakpoint adopted in this study (50, 51). For carbapenems, the *Tsukamurella* isolates were found to be slightly more frequently non-susceptible to imipenem by the broth microdilution method than by the disk diffusion method (Tables 3 and 5). All the *Gordonia* isolates were found to be susceptible to cephalosporins using the disk diffusion method, whereas *Tsukamurella* isolates showed varying degrees of non-susceptibility to this class of agents, with the highest susceptibility rate of 100% to cefepime (Table 5). The disk diffusion test confirmed tigecycline as an *in vitro* active agent against *Tsukamurella* and *Gordonia* isolates (Tables 3 to 5).

**TABLE 5:** Results of antimicrobial susceptibility testing using the disk diffusion method among *Tsukamurella* and *Gordonia* isolates according to species

Antibiotic disks, *N* (%) of non-susceptible isolates	*Tsukamurella* spp. (*n* = 15)	*T. tyrosinosolvens* (*n* = 9)	*T. pulmonis* (*n* = 3)	*T. inchonensis* (*n* = 2)	*T. sputi* (*n* = 1)	*Gordonia* spp. (*n* = 7)	*G. sputi* (*n* = 4)	*G. aichiensis* (*n* = 2)	*G. bronchialis* (*n* = 1)
Amoxicillin/clavulanic acid	15 (100)	9 (100)	3 (100)	2 (100)	1 (100)	0 (0)	0 (0)	0 (0)	0 (0)
Ceftriaxone	6 (40)	2 (22.22)	3 (100)	0 (0)	1 (100)	0 (0)	0 (0)	0 (0)	0 (0)
Chloramphenicol	1 5(100)	9 (100)	3 (100)	2 (100)	1 (100)	0 (0)	0 (0)	0 (0)	0 (0)
Cefotaxime	11 (73.33)	7 (77.78)	3 (100)	0 (0)	1 (100)	0 (0)	0 (0)	0 (0)	0 (0)
Cefepime	0 (0)	0 (0)	0 (0)	0 (0)	0 (0)	0 (0)	0 (0)	0 (0)	0 (0)
Cefoxitin	15 (100)	9 (100)	3 (100)	2 (100)	1 (100)	7 (100)	4 (100)	2 (100)	1 (100)
Linezolid	0 (0)	0 (0)	0 (0)	0 (0)	0 (0)	0 (0)	0 (0)	0 (0)	0 (0)
Vancomycin	0 (0)	0 (0)	0 (0)	0 (0)	0 (0)	0 (0)	0 (0)	0 (0)	0 (0)
Ertapenem	15 (100)	9 (100)	3 (100)	2 (100)	1 (100)	7 (100)	4 (100)	2 (100)	1 (100)
Tigecycline	0 (0)	0 (0)	0 (0)	0 (0)	0 (0)	0 (0)	0 (0)	0 (0)	0 (0)
Trimethoprim/sulfamethoxazole	0 (0)	0 (0)	0 (0)	0 (0)	0 (0)	0 (0)	0 (0)	0 (0)	0 (0)
Moxifloxacin	0 (0)	0 (0)	0 (0)	0 (0)	0 (0)	0 (0)	0 (0)	0 (0)	0 (0)
Imipenem	0 (0)	0 (0)	0 (0)	0 (0)	0 (0)	0 (0)	0 (0)	0 (0)	0 (0)
Ciprofloxacin	0 (0)	0 (0)	0 (0)	0 (0)	0 (0)	0 (0)	0 (0)	0 (0)	0 (0)
Minocycline	0 (0)	0 (0)	0 (0)	0 (0)	0 (0)	0 (0)	0 (0)	0 (0)	0 (0)

### Mutation within *gyrA*

The quinolone-resistance-determining region (QRDR) of the *gyrA* gene in *Tsukamurella* species was determined to stretch from amino acids 75 to 114 by multiple-sequence alignment with NCBI reference sequence of *M. tuberculosis* H37Rv (NP_214520.1) (52). In this study, a unique mutation S91R located within the QRDR of the *gyrA* gene occurred in the quinolone-resistant isolate of 22TM00764. It may be implicated that this DNA gyrase subunit substitution in 22TM00764 conferred high resistance to levofloxacin and ciprofloxacin but intermediate to moxifloxacin. We considered that this mutation in *Tsukamurella* species demonstrated identical functionality with the corresponding A90V mutation in *the gyrA* gene of *M. tuberculosis* H37Rv (NP_214520.1), which was the second most frequent mutation associated with lower level quinolone resistance and moxifloxacin effectiveness (M24-A3) (42).

## DISCUSSION

*Tsukamurella* species are rare but emerging pathogens causing human infections at different body sites, with an underestimated prevalence of pulmonary infection because they share remarkable similar clinical features with other mycolic acid–containing genera of the order Actinomycetales, especially *M. tuberculosis* (1, 3). In this context, the understanding of this rare infectious agent is needed to find methods for accurate and rapid identification, to better understand its epidemiology within hospitals, to increase knowledge of its clinical, microbiological and molecular characteristics, to establish specific criteria for susceptibility testing, and to investigate its mechanisms of resistance, which would otherwise remain fragmentary and incomplete. This study was novel in exploring the infections caused by various *Tsukamurella* species in China and aimed to elaborate their characteristics to improve the understanding of the clinical, microbiological, and molecular aspects, thus covering species distribution, antimicrobial susceptibilities, and quinolone resistance mechanism of *Tsukamurella* infections.

Considering that *Tsukamurella* lung disease manifests as tuberculosis-like syndromes (18), our study may be conceivable that pulmonary infection caused by *Tsukamurella* has been underestimated in China, the world’s second-largest country with a high tuberculosis burden (53). Among the 10 cases of pulmonary infection, *T. inchonensis* and *T. sputi* were described for the first time as potential pathogens of pulmonary infections (15). In this study, reinfection by another *Tsukamurella* species was observed in a patient with immunoglobulin G (IgG4)-related diseases for more than 3 years. Immunodeficiency and chronic pulmonary disease appeared to be risk factors for *Tsukamurella* pneumonia; the presence of bronchiectasis and pulmonary nodules on imaging was also highly correlated with this infection. Cases of pulmonary *Tsukamurella* infection were primarily diagnosed as mycobacterial infection, with extreme side effects to empirical anti-tuberculosis therapy (13). As anti-tuberculosis therapy is usually of prolonged duration, various adverse drug reactions may cause associated morbidity and even mortality if not recognized early (54). In our study, 5 out of 10 patients were suspected of having tuberculosis without isolation of mycobacterium tuberculosis. Of these, one patient was misdiagnosed as having tuberculosis due to the detection of acid-fast bacilli in a sputum smear, similar to a clinical case reported by Liu et al. (19), and three patients were receiving anti-tuberculosis therapy at the outset. Thus, an effective identification method is needed to develop an accurate diagnosis of *Tsukamurella* infections. Furthermore, six patients with *Tsukamurella* infection had good clinical outcomes after treatment with quinolones or sulfonamides. We also encountered a case of CR-BSI caused by *T. tyrosinosolvens* in a patient with underlying cancer who was immunosuppressed and underwent surgery within 30 days. She recovered after removal of the infected line and received antibiotic treatment of ciprofloxacin and amikacin. The combination of catheter removal and appropriate antibiotic treatment is considered an essential therapeutic method for the treatment of catheter-related infections caused by *Tsukamurella* species (55, 56).

The phenotypic similarities of *Tsukamurella*, *Gordonia*, *Rhodococcus*, *Actinomyces*, *Nocardia*, and *Mycobacterium* have resulted in unreliable identification using phenotypic or commercially available automated systems (18). MALDI-TOF MS has been employed as a user-friendly, rapid, and cost-effective means of identifying bacteria, fungi, and mycobacteria in routine clinical laboratories (57). However, except for 2 isolates of *T. inchonensis*, the other 20 isolates were all misidentified, including species-level misidentification of 13 *Tsukamurella* isolates and genus-level misidentification of 7 *Gordonia* isolates under the MALDI-TOF MS platform in our routine study. Teng et al. reported that none of the 60 isolates belonging to five different *Tsukamurella* species were correctly identified at the species level by MALDI-TOF MS with the original Bruker database V.6.0.0.0 (29). However, after optimizing the database by adding, the mass spectrum profiles of all 11 currently recognized *Tsukamurella* species as a reference; 59 of the 60 isolates were correctly identified to the species level (29). Therefore, once the database has been optimized by adding more Gram-positive rods, MALDI-TOF MS may serve as a beneficial tool for the routine identification of *Tsukamurella* species in clinical microbiology laboratories (1, 29, 58). Although sequence-based identification is considered to be a comparatively rapid and reliable molecular technique for the identification of *Tsukamurella* species (41), it has not yet been adopted as a routine identification method in clinical microbiology laboratories because it is expensive, time-consuming, and technically demanding for this purpose (29). Consistent with previous studies (37, 41, 59), this study also suggested that 16S rRNA gene sequencing was insufficient to discriminate among *Tsukamurella* spp. but was enough to discriminate *Tsukamurella* and *Gordonia*. Four additional housekeeping genes, *rpoB*, *secA*, *ssrA*, and *groEL*, were successfully used in this study for species-level identification in 16 and 12 closely related species of the genus *Tsukamurella*, respectively. Contrary to previously recommended target genes of *groEL* and *secA* (37, 41), we confirmed the identification performance of these two genes while also demonstrating the efficacy of the *ssrA* gene as a promising tool. The *ssrA* gene exhibited the highest intraspecies discrimination but the shortest sequence length in the 12 closely related species of the genus *Tsukamurella*. Nevertheless, the inclusion of more sequences from the four novel *Tsukamurella* species, including *T. asaccharolytica*, *T. hominis*, *T. conjunctivitidis*, and *T. ocularis*, may further help evaluate the efficacy of this gene for *Tsukamurella* identification.

Microbiological morphology is unreliable for accurate identification of species from the order Actinomycetales, including *Tsukamurella* and *Gordonia* (18). However, the phenotypic characteristics could remind us when the morphology did not match the identification result in the clinical laboratory or a simple distinction was made for different genera by rapid staining. In terms of phenotypic characteristics, for example, *Tsukamurella* and *Gordonia* isolates grew well on Columbia blood agar plates at 37°C for 48 h. This rapid growth characteristic was completely different from that of *M. tuberculosis* and nontuberculous mycobacteria (60). Overall, compared with the colony appearance of *Gordonia* species, *Tsukamurella* species were generally bigger, drier, wrinkled, and resembled a rough membrane with irregular spreading fringes. *Tsukamurella* and *Gordonia* species are both Gram-positive bacilli and are difficult to distinguish by Gram staining. However, it appears that *Tsukamurella* isolates are slender rod-shaped bacilli, whereas *Gordonia* isolates are short and thick cocci-rod bacilli. Furthermore, positive modified Ziehl–Neelsen staining and partially positive Ziehl–Neelsen staining were clearly observed in *Tsukamurella* isolates but not in any of the *Gordonia* isolates. Therefore, tuberculosis cannot be diagnosed by the detection of acid-fast bacilli alone, a lesson to be learned from the misdiagnosis of case 10 in our study and the previously described case (19).

The optimal management of *Tsukamurella* infections remains to be determined because of the paucity of antimicrobial susceptibility for this genus. This study revealed the *in vitro* antimicrobial susceptibility profiles of 34 antibiotics for *Tsukamurella* and *Gordonia* isolates using the microbroth dilution method. Previous studies have shown that *Tsukamurella* isolates are resistant to penicillin, cefoxitin, and cephalosporin, which are commonly prescribed for the treatment of nontuberculous mycobacterial infections (5, 38, 46). However, *Tsukamurella* isolates in this study were susceptible to the third- and fourth-generation cephalosporins. The differentiate antimicrobial susceptibility to amoxicillin/clavulanic acid (2:1) provided an additional method to discriminate between *Tsukamurella* and *Gordonia* species in terms of drug susceptibility. Previous studies summarized that *Tsukamurella* isolates were susceptible to aminoglycosides, macrolides, and vancomycin (46, 56), but these antibiotics were not included in our recommended list without susceptibility testing, except for amikacin. Regarding anti-tuberculosis agents, rifampin had probable efficacy, but isoniazid, ethionamide, and ethambutol were completely ineffective in terms of *in vitro* susceptibilities against *Tsukamurella* and *Gordonia* isolates. Based on our results, quinolones, trimethoprim/sulfamethoxazole, amikacin, minocycline, linezolid, and tigecycline showed higher *in vitro* susceptibilities against *Tsukamurella* and *Gordonia* isolates. Although one *T. tyrosinosolvens* isolate in this study was not susceptible to quinolones, we still recommended quinolones as first-line or empirical therapy in *Tsukamurella* infection because it was commonly confused with tuberculosis (13, 19). However, a favorable prognosis would be achieved by individualized treatment based on identification of the cause of infection and antimicrobial susceptibilities. Sulfonamides were good choice for the treatment *Tsukamurella* pulmonary infections with co-isolation of *P. jirovecii* (61), but not with that of *P. aeruginosa* which is intrinsically resistant to sulfonamides (51, 62). Our data may help to establish exclusive breakpoints for *Tsukamurella* species, even for the order Actinomycetales. Notably, we proposed that the S91R substitution within the QRDR of the *gyrA* gene could confer high resistance to levofloxacin and ciprofloxacin and intermediate to moxifloxacin. Chauffour et al. concluded that mutations in residues at positions 87, 89, 91, and 92 in the QRDR of the *gyrA* gene confer resistance by impacting the binding of the quinolones to mycobacterial cells (52).

AST using the disk diffusion method is easy, universal, and convenient for most clinical laboratories. However, this study found disk diffusion to be an inappropriate method for testing ciprofloxacin, imipenem, and minocycline, based on the abnormal results of undetected non-susceptible isolates. For the other antimicrobials in this study, disk diffusion testing could be used to test the susceptibilities of *Tsukamurella* and *Gordonia* isolates after the clinical standard breakpoint has been established.

This study was undertaken because of the confusion between *Tsukamurella* and other mycolic acid–containing genera of the order Actinomycetales and the need for accurate identification methods to avoid misdiagnosis in clinical settings. To the best of our knowledge, we have investigated the susceptibilities of four species of *Tsukamurella* to the widest range of antimicrobial agents, as well as neatly compared the susceptibility discrimination between *Tsukamurella* and *Gordonia*. For the first time, we described *T. inchonensis* and *T. sputi* as potential pulmonary pathogens and elucidated a quinolone resistance mechanism in *T. tyrosinosolvens*. However, there are several limitations in this study. Firstly, the pathogenicity of pulmonary infections caused by *Tsukamurella* species is not completely certain, as they were all isolated from sputum samples. In addition, although the point mutation of the *gyrA* gene was found to confer quinolone resistance in *T. tyrosinosolvens*, this needs to be confirmed by further studies with phenotypic and genotypic experiments. Finally, the identification efficiency of the MALDI-TOF MS system for this rare pathogen should be further evaluated by establishing an in-house database.

In conclusion, this study highlighted the emergence of *Tsukamurella* species as a potential pathogen mainly for pulmonary infections in patients with various risk factors. The molecular methods accurately identified this pathogen to avoid misdiagnosis as pulmonary tuberculosis. We confirmed that *groEL* and *secA* genes are reliable for identifying *Tsukamurella* species. Additionally, we proposed the *ssrA* gene as a promising tool with the highest intraspecies discrimination but the shortest sequence length. This study remarkably supported quinolones, trimethoprim/sulfamethoxazole, amikacin, minocycline, linezolid, and tigecycline with potent *in vitro* activity against *Tsukamurella* isolates. This study was novel in elucidating the mechanism conferring quinolone resistance in *T. tyrosinosolvens*.

## SUPPLEMENTAL MATERIAL

Supplemental data spectrum.01644-23-s0001.docx Tables S1 and S2.

ASM does not own the copyrights to Supplemental Material that may be linked to, or accessed through, an article. The authors have granted ASM a non-exclusive, world-wide license to publish the Supplemental Material files. Please contact the corresponding author directly for reuse.
