# Supplementary material for: Systematic investigation of the emerging pathogen of Tsukamurella species in a Chinese tertiary teaching hospital
Source: Microbiol Spectr. 2023 Oct 24;11(6):e01644-23. doi: 10.1128/spectrum.01644-23 (PMC10715089; doi:10.1128/spectrum.01644-23)
Supplement: Supplemental data — Tables S1 and S2. [file spectrum.01644-23-s0001.docx]

**SUPPLEMENTARY DATA**

**TABLE S1. Antibiotic disks used for antimicrobial susceptibility testing and interpretation thresholds.**

| **Antibiotic** | **Dose/disk (µg)** | **Interpretation threshold (mm)*** | | |
| --- | --- | --- | --- | --- |
|  |  | **S** | **I** | **R** |
| Amoxicillin/clavulanic acid | 20/10 | ≥23 | 16-22 | <16 |
| Ceftriaxone | 30 | ≥26 | 23-25 | <23 |
| Chloramphenicol | 30 | ≥18 | 13-17 | <12 |
| Cefotaxime | 30 | ≥26 | 23-25 | <23 |
| Cefepime | 30 | ≥21 | 19-20 | <19 |
| Cefoxitin | 30 | ≥22 | − | ≤21 |
| Linezolid | 30 | ≥28 | 24-27 | <24 |
| Vancomycin | 30 | ≥17 | − | − |
| Ertapenem | 10 | ≥28 | 26-27 | <26 |
| Tigecycline | 15 | ≥22 | − | <22 |
| Trimethoprim / Sulfamethoxazole | 25 | ≥16 | 10-15 | <10 |
| Moxifloxacin | 5 | ≥24 | 21-23 | <21 |
| Imipenem | 10 | ≥24 | 17-23 | <17 |
| Ciprofloxacin | 5 | ≥25 | 22-24 | <22 |
| Minocycline | 30 | ≥19 | 17-18 | <17 |

NOTE: S: susceptible, I: Intermediate, R: resistant. The interpretation thresholds for chloramphenicol and cefoxitin are adapted from guidelines of the Clinical and Laboratory Standards Institute (CLSI) devoted to *Staphylococcus aureus*(1). The interpretation thresholds for the other antibiotic disk are adapted from guidelines of the Antibiogram Committee of the French Society of Microbiology (CA-SFM) devoted to gram positive bacteria (2).

**TABLE S2. Identification results by MALDI-TOF MS and 16S rRNA sequencing for *Gordonia* isolates**

| **Strain no.** | **Site of isolation** | **Initially identification by MALDI-TOF MS** | **16S rRNA sequencing** | **Accession numbers** |
| --- | --- | --- | --- | --- |
| 21NC09120 | Sputum | *T. pulmonis* | *G. sputi* | OP799739 |
| 21NC05575 | Sputum | *T. pulmonis* | *G. aichiensis* | OP799741 |
| 21NC05485 | Sputum | *Tsukamurella spp* | *G. sputi* | OP799742 |
| 21NC03853 | Sputum | *T. inchonensis* | *G. sputi* | OP799743 |
| 21NC03853 | Sputum | *T. pulmonis* | *G. aichiensis* | OP799744 |
| 21NC03851 | Sputum | *T. pulmonis* | *G. sputi* | OP799745 |
| 19W04084 | Hip fat necrosis | *T. pulmonis* | *G. bronchialis* | OP799746 |

MALDI-TOF MS, Matrix-assisted laser desorption ionization–time-of-flight mass spectrometry, 16S rRNA, 16S ribosomal DNA.

**SUPPLEMENTARY REFERENCE:**

1. https://www.resapath.anses.fr/resapath_uploadfiles/files/Documents/2013_CASFM. pdf.

2. CLSI. Performance Standards for Antimicrobial Susceptibility Testing. 26th ed. CLSI supplement M100S. Wayne, PA: Clinical and Laboratory Standards Institute; 2016.
